# Breast cancer diagnosed after age 70 years in Israeli *BRCA1*/*BRCA2* pathogenic sequence variant carriers: a single institution experience

**DOI:** 10.1007/s10549-023-07234-1

**Published:** 2024-02-21

**Authors:** Hila Bufman, Renata Faermann, Osnat Halshtok-Neiman, Anat Shalmon, Michael Gotlieb, David Samoocha, Yael Yagil, Dana Madorsky Feldman, Eitan Friedman, Miri Sklair-Levy

**Affiliations:** 1 The Institute of Radiology, Sheba Medical Center, 1 Emek Ha-Ella St, 5265601 Tel-Hashomer, Israel; 2 The Meirav High Risk Clinic, Sheba Medical Center, Tel-Hashomer, Israel; 3 The School of Medicine, Tel Aviv University, Tel Aviv, Israel; 4 The Center for Personalized Medicine, Assuta Medical Center, Tel Aviv, Israel

**Keywords:** *BRCA* PSV carriers, Breast cancer risk, Early detection, Age limit for breast cancer surveillance

## Abstract

**Purpose:**

A semi-annual surveillance scheme from age 25 to 30 years is offered to *BRCA1*/*BRCA2* pathogenic sequence variants (PSVs) carriers for early detection of breast cancer (BC). There is a paucity of data on the yield of adhering to this scheme beyond 70 years of age.

**Methods:**

Female *BRCA1*/*BRCA2* PSV carriers followed at the Meirav high-risk clinic, Sheba Medical center, Israel were eligible. Type and frequencies if use of Imaging modalities, breast biopsies and histological outcomes for participants after age 70 years were retrieved and analyzed.

**Results:**

Overall, the study encompassed 88 consenting participants (46 *BRCA1* carriers) mean age ± SD 73.7 ± 3.3 years (range 70–90 years), followed for an average of 3.8 years (range 1–11 years). Ten carriers (11.3%) were diagnosed with BC after age 70 years (mean age at diagnosis 72 ± 2 years) and an additional case was diagnosed with breast lymphoma. The imaging modality that has led to most diagnoses was MRI (8/11 cases). Eight of these ten cases were previously diagnosed with BC prior to age 70 and in six, BC past 70 years was in the contralateral breast. The lesions size averaged 1.29 ± 0.75 cm, with IDC and DCIS diagnosed in five cases each, and none had lymph node involvement.

**Conclusion:**

In ~10% of *BRCA1*/*BRCA2* PSV carriers BC is diagnosed by breast imaging after age 70 years. If these results are validated in a larger study, the guidelines for the maximum age for BC surveillance in high risk women should be revisited and set at 75 years.

## Introduction

Women harboring *BRCA1* or *BRCA2* (*BRCA*) germline pathogenic sequence variants (PSVs) are at a substantially increased lifetime risk for developing breast cancer (BC) estimated at 72% and 69%, respectively [1], at times diagnosed at an early age—< 40 years. These high risk women are offered a surveillance scheme aimed at early detection of BC from age 25 to 30 years that is based on semiannual breast exam and breast imaging: mammograms alternating with breast MRIs [2]. While such a surveillance scheme is well established and is widely practiced worldwide in specialized high-risk clinics, there is no consensus or guidelines as to the upper limit of age above which such screening scheme should no longer be offered or practiced, and such an age limit, where it exists, varies by country and recommending body [3]. In a comprehensive analysis of the practices in several prominent countries, Madorsky-Feldman et al. [4] reported that maximal age for MRI and BC surveillance varies from 69 to 80 years among surveyed countries, and most countries do not have an upper age limit of BC surveillance. Specifically, in Germany the upper age limit for *BRCA* carriers is 70 years [5]. Boddicker and co-workers [6] showed that *BRCA* PSV carriers over age 65 years continue to be at increased BC risk, with an estimated residual risk of BC from age 66 to 85 years 18–20% (compared with 6.8% in the general population), with an odds ratios (OR) of 3.37 and 2.62 for *BRCA1* and *BRCA2*, respectively. These data suggest considering continued MRI screening beyond age 70 years in *BRCA* PSV carriers.

The current study aimed at evaluating the yield of semi-annual BC screening in Israeli *BRCA* carriers followed in a single high-risk clinic, by analyzing the BC rates and histological features diagnosed in these women above 70 years of age.

## Materials and methods

The Meirav high-risk clinic at the Sheba Medical center, Tel-Hashomer, Israel is a dedicated female *BRCA* carriers’ clinic that has been operative since 2007. The surveillance scheme offered at this high-risk clinic follows mostly the American Cancer Society—ACS (https://www.cancer.org/health-care-professionals/american-cancer-society-prevention-early-detection-guidelines.html) and National Comprehensive Cancer Network NCCN (https://www.nccn.org/) guidelines. Briefly, all *BRCA* PSV carriers undergo biannual clinical breast examination from age 25 years, annual breast MRI starting at age 25 years, alternating with annual mammography and sonography starting at age 35 years. Self-developed guidelines also include clinical breast examination and sonography every 3 months in pregnant and breastfeeding *BRCA1*/*BRCA2 *PSV carriers. All registered *BRCA1*/*BRCA2 *PSV carriers attending the Meirav high-risk clinic who were at or above age 70 years at any point in their semi-annual clinic visit were included in this study. Carriers who underwent bilateral risk reducing mastectomy (BRRM) were excluded from the study. The study was approved by the ethics committee of the Sheba Medical Center, and given its retrospective nature and lack of direct patient contact, was exempt from obtaining participants’ specific written informed consent. Notably, all participants consented for the initial *BRCA *testing and data acquisition as part of the Oncogenetics counseling process and ongoing surveillance. Data were reviewed for all cases attending the clinic from January 2009 to January 2022, and included date of birth, type and location of the specific *BRCA1* or *BRCA2* PSV, previous breast malignancy, year at joining the clinic and number of visits. In addition, number of mammographs, ultrasonograms (US), and MRIs performed during follow up, number and pathological results of all breast biopsies and the imaging modality used for obtaining the biopsy as well as the imaging modality that has shown an abnormality leading to biopsy, and recording other, non-breast malignancies, risk reducing salpingo-oophorectomy, and age of that procedure. For those diagnosed with BC or non-breast malignancy during follow up and over the age of 70 years, the following additional data were collected: presenting symptoms, histopathological features of the breast tumor, lesion size and location, BIRADS score, and type of surgery.

Statistical analysis, mean and standard deviations of all relevant parameters for the descriptive statistical analyses were calculated by Student’s *t* test and chi square by using SPSS Statistics software (version 27.0, IBM).

## Results

*Cohort characteristics and follow up*—A total of 97 *BRCA1*/*BRCA2* PSV carriers who were at or over age 70 years during any time of their participation of the surveillance scheme offered at the Meirav high risk clinic between 2009 and 2022 were identified. Of these women, nine were excluded due to lack of pertinent medical data relevant to this study, thus leaving a final cohort of 88 participants: 46 (52.2%) were *BRCA1* PSV carriers and the rest (47.8%)—carried a *BRCA2* PSV. Most PSVs were one of the three predominant PSVs in Jewish Ashkenazi women—*BRCA1* {c.5266dupC (p.Gln1756Profs) [5382insC], c.66_67AG (p.Glu23fs) [185delAG]} and *BRCA2* {c.5946del (p.Ser1982fs) [6174delT]}. Mean age of participants was 73.7 ± 3.3 years (range 70–90 years). Of participants 48/88 (54.5%) had BC diagnosed prior to age 70 years, mean age at first BC diagnosis was 57.7 ± 10.6 years, 47.9% (23/48) of these cases were *BRCA1* carriers, 50% (24/48) are *BRCA2* carriers, with data on the precise mutant gene not available in one case. Most study participants (77–87.5%) underwent risk reducing salpingo-oohphrectomy at and median age of 43 years (range 37–56 years). The cumulative person-years of follow up was 334 years, with a mean of 3.8 years per participant. Mean number of mammograms during follow up was 3 (range 0–12), MRIs—3.6 (range 0–10), and imaging guided biopsies—0.4 (range 0–4).

*Cancer diagnoses at follow up*—Of 88 study participants, 10 were diagnosed with BC during the follow up at or after age 70 years (11.36%), and an additional participant was diagnosed with lymphoma of the breast (age 72). The following analysis is restricted only to those diagnosed with BC and excluding the breast lymphoma case. In the ten BC cases, mean age at diagnosis was 72.3 ± 2 years; five were IDC and 5—DCIS. The lesions had a mean diameter of 1.29 ± 0.75 cm (range 0.5–2.8 cm) and none of the patients diagnosed with breast malignancy presented either with lymph node involvement or with distant metastasis. All but three cases had no palpable mass, and the most common diagnostic imaging modality was MRI (8/11). The mean number of mammograms during follow up was 4.5 (range 2–10), the mean number of MRIs was 6.1 (range 1–9), the mean number of imaging guided biopsies was 1.7 (range 1–4). The most frequent modality of diagnosis was MRI (8/11), followed by US (2/11) and mammography (1/11). As to the modality of imaging guided biopsy, 5/11 patients underwent US guided biopsy, and same number of patients underwent MRI guided biopsy (45.5% each), only one patient underwent stereotactic core biopsy (9%).

Of the ten cases of breast malignancy diagnosed above age 70 years, eight had previous breast malignancy at 3–41 years prior to the current diagnosis of BC and invariably were all deemed to be disease free at the start of the study. Furthermore, in 6/8 cases, the current breast malignancy was in the contralateral breast to the previously diagnosed BC.

The calculated incidence of BC in our cohort (= observed) is 946/100,000 women. The age standardized rate (ASR) for BC in the Jewish general population in the 70–74 age group (= expected) is 420.05/100,000 in Israel (https://www.health.gov.il/UNITSOFFICE/HD/ICDC/ICR/), a statistically significant difference (*p* = 0.0001), the relative risk (RR) is 2.53 (95% CI 2.25–2.86) *p* < 0.0001. Relevant clinical and pathological details are provided in Table 1.

**Table 1:** Features of breast malignancies diagnosed during follow up

No	BRCA1/BRCA2	Specific mutation	Age at Dx	Diagnosing modality	Biopsy modality	Pathology	Contralateral/ipsilateral to previous malignancy	Tumor size (cm)	Surgery type	ER	PR	HER2	KI-67
1	BRCA2	6174delT	75	MRI	MRI	DCIS	Contralateral	2.3	Lumpectomy	+	+		
2	BRCA1	185delAG	75	US	US	IDC	Contralateral	0.7	Lumpectomy	−	−	+	IM
3	BRCA2	6174delT	74	MRI	US	IDC	Ipsilateral	1.5	Lumpectomy	+	−	−	Low
4	BRCA2	6174delT	72	US	US	Lymphoma							
5	BRCA1	Y978X	71	Mamo	Stereotactic	DCIS	Contralateral	2.8	Lumpectomy	+	+	−	
6	BRCA1	5382insC	74	MRI	MRI	DCIS	Contralateral	1	Lumpectomy	−	−		
7	BRCA1	185delAG	70	MRI	MRI	IDC		0.5	Lumpectomy	−	−	−	High
8	BRCA2	6174delT	70	MRI	MRI	DCIS	Contralateral	1.5	Lumpectomy	−	−		
9	BRCA1	185delAG	73	MRI	US	IDC		0.6	Lumpectomy	+	−	−	High
10	BRCA1	Y978X	71	MRI	MRI	DCIS	Contralateral	1.2	Lumpectomy	+	+		
11	BRCA1	5382InsC	70	MRI	US	IDC	Ipsilateral	0.8	Mastectomy	−	+	−	High

*MRI* magnetic resonance imaging, *US* ultrasonography, *Mam0* mammography, *DCIS* ductal carcinoma in situ, *IDC* invasive ductal carcinoma, *ER* estrogen receptor, *PR* progesterone receptor, *HER2 *human epidermal growth factor receptor 2, *IM* intermediate

## Discussion

In the current study, ~10% of female *BRCA1*/*BRCA2* PSV carriers developed BC after age 70 years but none over 75 years of age. These findings may have clinical implications. First, it supports the notion that for women in the 70–75 year age group, being a *BRCA* carrier is still a risk factor for developing BC, and is associated with at least a doubling of the BC risk compared with non-carriers [7]. While lifetime risks for BC in *BRCA* mutation carriers are up to seven times that of the general population [8], these increased risks are not linear curve and are most pronounced in the younger age groups—i.e., under 55 years of age. Thus, these younger, higher risk women are offered the tight surveillance scheme aimed at early BC detection that requires a semiannual physical exam and breast imaging [9, 10]. The uncertainty to what extent this scheme is still applicable for more advanced age *BRCA* carriers is reflected in part in the lack of consensus to the upper limit of the age at which such a heightened surveillance scheme should be continued ranging from 69 years to no age limit [3]. Based on the results of the current study and the study by Boddicker et al. [6], and pending validation of the results presented herein, the upper age limit should be minimally set at 75 years of age and not earlier. Indeed, the statistically significant difference between observed:expected rates of BC in that population and the RR that is ~2.5 compared with age adjusted average risk population, further support the notion that these women should still be offered a BC surveillance scheme. Furthermore, previous studies that focused on penetrance of BRCA PSVs in the older age group seem to support the results of the present study. Stjepanovic and coworkers [11] report that the cumulative BC rate and annual incidence rate in BRCA1 PSV carriers (*n* = 463) age 60–80 years followed up for an average of 7/9 years were 20.1% and 1.8% and for BRCA2 PSV carriers (*n* = 236) these rates were 17.3% and 1.7%, respectively. Another study [12] analyzing women diagnosed with triple-negative BC above age 61 years (*n* = 130), reported that of six *BRCA1* and five *BRCA2* PSV carriers, three were diagnosed above age 70. Interestingly one of those did carry the predominant Jewish mutation in BRCA1 c.68_69del (AKA 185delAG). In a study that encompassed 69 BRCA carriers who were at least 75 years of age (14% Ashkenazi Jews) only 6 women were cancer free up to that age and of the 4 women who developed new cancer diagnosis after age 75 (all pancreatic cancer) 3 had pre 70 diagnosis of either BC or OvC. Notably none of the women who survived to be 90 years of age developed novel BRCA related cancer after age 75 years [13].

Most cancers in the current study were diagnosed primarily by breast MRI at a relatively early stage, of up to T1N0M0 [11], none of the BC diagnosed cases had positive lymph nodes, and the average size for IDC was 1.29 cm—a size that is assigned “small size” by international criteria (T1) [14]. Thus, detecting BC at these early stages and the small sized tumor, in all likelihood, minimized the need for an aggressive chemotherapy treatment. This should be taken into account when considering cost-effectiveness of BC screening by MRI in this population. Some national guidelines [e.g., NICE (https://www.nice.org.uk/guidance/cg164/chapter/Recommendations (accessed on 5 June 2022), INCa (https://www.e-cancer.fr/Expertises-et-publications/Catalogue-des-publications/Femmes-porteuses-d-une-mutation-de-BRCA1-ou-BRCA2-Detection-precoce-du-cancer-du-sein-et-des-annexes-et-strategies-de-reduction-du-risque (accessed on 5 July 2022), SEOM [15], Belgian society of human genetics (http://www.college-genetics.be/fr/pour-les-professionnels/recommandations-et-bonnes-pratiques/guidelines.html (accessed on 5 July 2022)] suggest stopping use of MRI screening in *BRCA* PSV carriers at ages 65–70 years, given that the risk in the older age group is significantly lower compared with younger carriers, and recommend mammography as the only breast imaging modality that should be offered routinely. If the results of the current study are validated in a larger prospective study, these recommendations for the sole use of mammography as a breast imaging modality for older *BRCA* PSV carriers should be re considered.

This study has several limitations. First, this was a retrospective study, and the results should be interpreted cautiously, given the inherent limitations of a retrospective study design. Second, there was no internal control group, and BC incidence was compared to the reported incidence from the literature. Finally, the study cohort was relatively small as we included only *BRCA* carriers who adhered to BC screening scheme after age 70 years.

## Conclusion

BC incidence in Israeli *BRCA* PSV carriers above the age of 70 was higher in our cohort compared to the expected rate in the general Israeli population, all detected cancers were small with no lymph node involvement and mostly detected by MRI. If these results are confirmed and validated, it might be appropriate to continue BC screening in *BRCA* PSV carriers primarily using MRI until the age of 75. Further prospective studies encompassing more carriers of diverse *BRCA* PSVs are urgently warranted.

## Data Availability

The data that formed the basis for this study are protected to ensure patient privacy by the IRB approval form.
